# Oxymatrine Protects TGFβ1-Induced Retinal Fibrosis in an Animal Model of Glaucoma

**DOI:** 10.3389/fmed.2021.750342

**Published:** 2022-02-18

**Authors:** Ashmita Das, Onkar Kashyap, Amrita Singh, Jaya Shree, Kamta P. Namdeo, Surendra H. Bodakhe

**Affiliations:** ^1^Department of Pharmacology, SLT Institute of Pharmaceutical Sciences, Guru Ghasidas Vishwavidyalaya (A Central University), Bilaspur, India; ^2^ISF College of Pharmacy, Moga, India; ^3^Rungta Institute of Pharmaceutical Sciences and Research, Bhilai, India

**Keywords:** oxymatrine (OMT), TGFβ1, steroid induced glaucoma, retinal pathology, lenticular alterations, biochemical estimations

## Abstract

Glaucoma has engulfed a huge population of the world into its claws of blindness as it remains asymptomatic until nearly 40% of the neurons are lost and the only option left is for patients to be subjected to symptomatic treatments or surgical methods, neither of which is completely effective in curing the disease as they do not restore the physiological dimensions at the neuronal level. Among the several factors that drive the pathophysiology of glaucoma, one is the involvement of fibrogenic factors, such as transforming growth factor β (TGFβ) which remodels the extracellular matrix (ECM) and, thus, the deposition of fibrotic material in the retina, resulting in the progression of primary open-angle glaucoma (POAG). The primary objectives of this study were to evaluate the protective effects of oxymatrine (OMT) in the steroid-induced glaucoma model in experimental rats and to determine the role of transforming growth factor β1 (TGFβ1) in the pathogenesis of glaucoma and its consequent inhibition due to the antioxidant and the antiinflammatory, and also the TGFβ1 antagonistic, behavior of OMT. To that end, we experimentally elucidated the role of OMT, a TGFβ1 antagonist, that is known to play antiinflammatory and antioxidant roles in the steroid-induced glaucoma model in experimental rats, and using the enzyme-linked immunosorbent assay (ELISA), we observed a direct inhibitory effect of OMT on the pathogenesis of glaucoma. The antioxidant and the antiinflammatory potentials of OMT were determined using several biochemical methods to determine the major antioxidants in the retinal layers, such as superoxide dismutase (SOD), glutathione peroxidase (GP_X_), catalase (CAT), and glutathione (GSH), along with the nitrite and the malondialdehyde (MDA) concentration levels. As a result, OMT was found to reduce the total protein content in the retinal layers, a correlation that has not been previously reported. Moreover, the impacts of OMT on the major governing ATPases, namely Na^+^/K^+^ ATPase and Ca^2+^ATPase, along with its impacts on the intracellular ionic concentrations of Na^+^, K^+^, and Ca^2+^, were determined and were found to point toward OMT, restoring homeostasis in glaucomatous animals. A clearer picture of the changes during the treatment was obtained using retinal images of the live animals and of the lenticular changes in the sacrificed animal; these images provided data on the pathological pathways leading to glaucoma inception and its consequent inhibition by OMT. The data reported in this study clearly indicate that OMT has a possible role in inhibiting the pathogenesis of glaucoma, and the data also permit the quantification of several biochemical parameters of concern.

## Introduction

Fibrotic disorders have been of increasing concern in the domain of ocular disorders, and the transforming growth factor β (TGFβ) plays a pivotal role in their progression. This multifunctional cytokine has been found to have a strong impact on the deposition and the expression of the extracellular matrix (ECM) and has been observed to be increased in aqueous humor (AH) and reactive optic nerve astrocytes of patients with primary open-angle glaucoma (POAG). TGFβ is a key player contributing to structural changes in the ECM of the trabecular meshwork (TM) and the optic nerve head (ONH), as characteristically seen in POAG, and TGFβ has a role in the production of protease inhibitors that hinder ECM degradation and cause an abnormal deposition of connective tissue that marks the onset of fibrotic disease (1–6). TGFβ1 has also been found to be elevated in the AH, along with TGFβ2, of patients with clinically active glaucoma and has also been found to be involved with the activation of thrombospondin-I *via* induction through dexamethasone (DEX) in TM cells, resulting in stress conditions (7). Moreover, TGFβ-mediated retinal fibrosis has been found to lead to an escalation in the intraocular pressure (IOP), which then causes pressure to be generated in the optic nerve neurons, as a result of which retinal ganglion cells (RGCs) die (8–10).

In the ONH, transforming growth factor β2 (TGFβ2)-induced changes are contributable to mechanobiological changes in the optic nerve axons that impair axonal transport and neurotrophic supply, thus leading to their continuous degeneration. The increase in the IOP further adds mechanical stress and strain to optic nerve axons and accelerates degenerative changes (11). Even though a variety of experimental findings have demonstrated the effective contribution of TGFβ2 in patients with glaucoma, the relationship between glaucoma and TGFβ1 has needed further investigation, which has led to the use of OMT, a TGFβ1 antagonist extracted from the roots of *Sophora flavescens Ait*, belonging to the family *Fabaceae* and a known savior for patients with fibrotic diseases (12–14).

Oxymatrine (OMT) has been chosen for this study because various researches and a literature review revealed it to be a potent inhibitor of TGFβ, an ECM protein extensively found in the TM and ONH and involved in the fibrotic pathway in glaucoma. Its accumulation in the TM causes mechanobiological alterations and finally leads to an increase in the IOP, subsequently contributing to glaucoma (8–10). In addition, OMT inhibits the inducible nitric oxide synthase (iNOS) and the nuclear factor kappa B, which are two important mediators of the inflammatory pathway associated with glaucoma progression (15, 16). OMT has further been found to inhibit the role of tumor necrosis factor alpha (TNFα), which enhances neurodegeneration by acting as a pressure enhancer on RGCs (15, 16). Even though the role of OMT in glaucoma retardation has yet to be reported, we here report, for the first time, that OMT may retard the development and the progression of glaucoma by acting on targets such as TGFβ and TNFα.

## Methods

### Drug Solution and Dosing

Oxymatrine was obtained as a generous gift from Shaanxi Pioneer Biotech, China, whereas dorzolamide was purchased from Intas Pharmaceuticals Pvt. Ltd, India, and DEX from Alfa Aesar, Boston, USA. The other chemicals required for the biochemical analyses were purchased from various sources and were of standard analytical grade. The ELISA kit for TGFβ was purchased from Elabscience, Houston, TX, USA. A stock solution of 0.1% DEX was prepared by dissolving DEX in distilled water and filtering the solution in aseptic conditions. The resultant solution was administered to the animals four times daily for 21 days *via* the ophthalmic route at a dose of 100 μl or 0.1 ml per topical application (17). A stock solution of 1% OMT was prepared in distilled water, and consequent dilutions were made to produce solutions with 0.5 and 0.25% concentrations of OMT. The aseptically filtered solutions were administered to the animals once daily *via* the ophthalmic route at a dose of 50 μl per topical application for 21 days after the occurrence of DEX-induced glaucoma (18). All the ophthalmic solutions were prepared under aseptic conditions and finally filtered two times in an aseptic zone.

### Experimental Animals

Experiments were carried out on male and female Sprague-Dawley rats, 8 to 10 weeks of age and weighing from 100 to 120 g, obtained from Chakraborti Enterprise, Narkeldanga, Kolkata- 700011, India. Before experimentation, the procedures were formally reviewed and approved by the Institutional Animal Ethics Committee (IAEC) (register number: 994/GO/Re/S/06/CPCSEA and reference number: 275/Pharmacy/2020) as per the guidance of the Committee for the Purpose of Control and Supervision for Experiments on Animals (CPCSEA), Government of India.

### Experimental Design

Animals with normal IOPs and no visible ocular disturbances were chosen for the study and were randomly allotted to different experimental groups, with six animals in each group. During the experimental protocol, the animals in the normal group received distilled water at a dose of 100 μl, topical, for 15 days. Uveitic glaucoma was induced in animals by the administration of DEX (0.1 ml of 0.1% solution, topical, i.e., ophthalmic route, four times daily, for 21 consecutive days) (17). The experimental animals allotted for the treatment groups received OMT at doses of 50 μl of 0.25, 0.5, or 1% solution once daily for 15 days *via* the topical route of administration. The experimental animals in the standard group were administered 100 μl of 1% of dorzolamide daily for 15 days *via* the topical route (19).

### Evaluation of Intraocular Pressure

Before the inducing agent or the treatments were to be applied, we, with the help of a Tono-Pen tonometer (Reichert Technologies, Depew, USA), regularly checked the experimental rats for the degree of IOP generated in the eyes. To create a baseline, we checked all the rats for IOP before administration of the drugs; thereafter, we regularly checked the animals until the day of sacrifice (20).

### Determination of the Lenticular Opacity

Assessment of the lenticular opacity was conducted through a photographic technique where the isolated lenses were washed immediately in double-distilled water and placed on graph paper. Photographs of the lenses were then taken using a cell phone camera (Photron Universal, Maharashtra, India) (21).

### Picturization of the Retinal Images of the Experimental Rats

The experimental rats were exposed to anesthesia, and their retinas were picturized using fundoscopic examination with a Panoptic ophthalmoscope (Welch Allyn, Skaneateles Falls, USA), in which the retina was illuminated through the pupil (22).

### Isolation of the Retinas and the Lenses From the Experimental Rats

The experimental rats were sacrificed using a high dose of anesthesia, after which the lenses and retinas were isolated using a posterior approach, washed with cold saline, and dried on a filter paper. The weighed retinas were homogenized with a 0.1-M phosphate buffer in nine volumes and centrifuged at 15,000 rpm for 5 min at 2°C−8°C using a refrigerated centrifuge (23). The supernatant was separated and utilized for further biochemical analysis with the help of a UV-visible spectrophotometer (Shimatzu, Japan).

### Quantitative Estimates of the Antioxidants in the Retinal Layers

The concentrations of antioxidant, including CAT, SOD, GSH, and GP_X_, were spectrophotometrically estimated, and the levels of lipid-layer damage were quantified using the MDA content in the retinal layers. The method detailed by Sinha (1972) was adopted for the determination of the CAT activity, which was measured using the rate of decomposition of hydrogen peroxide (H_2_O_2_) to water (H_2_O) and was measured at 530 nm against a blank. The CAT activity was expressed as μM of H_2_O_2_consumed/min/mg protein (24). The activity of SOD in the biological samples was calculated based on photoinhibition of nitro blue tetrazolium (NBT). The enzymatic activity of SOD was recorded as U/mg, where one unit (U) of SOD describes the number of enzymes that have effectively reduced the photoreduction rate of NBT to 50% (25). The activity of the GPx was assayed using the methods described by Tappel (1978), which is a prime requisite for the catalyzed reduction in GSH into GSH and water in the presence of H_2_O_2_ and is expressed as μM of GSH oxidized/min/mg protein (26). Ellman's reagent was used to estimate the GSH level. The reaction between GSH and Ellman's reagent [(27), 5'-dithiobis (2-nitrobenzoic acid)] displays a yellow-colored product that was read at 412 nm spectrophotometrically (28). Lipid peroxidation was denoted as an analytical parameter for the damage inherited by the lipid bilayer and was quantified using the MDA content in the sample. The method defined by Ohkawa et al. (29) was used to estimate MDA levels during the course of its reaction with thiobarbituric acid (TBA). The consequent reaction between TBA and MDA produced a colored, stable chromogen product that was measured at 532 nm. The MDA content was expressed as nM/mg protein (29).

### Estimates of Nitrite Levels

Assessment of the nitrite content in the retinal samples was performed following the method described by Green et al. (30). Sodium nitrite was utilized for the preparation of the standard curve. Nitrite content was expressed in nmoles/mg (30).

### Quantitative Estimates of the Total Protein Content in the Retinal Layers

The total protein content in the retinal lysate was measured using the procedure penned down by Lowry et al. (31).

### Quantitative Estimates of the Na^+^/K^+^ Atpase and the Ca^2+^Atpase Activities in the Retinal Layers

The Na^+^/K^+^ ATPase and Ca^2+^ATPase activities of the retina were quantified by employing the method of Manikandan et al. (32).

### Estimates of the Ionic Contents

Isolated retinas were washed with cold distilled water and homogenized (1 % w/v) with distilled water. The homogenate was filtered, and the ionic contents were estimated. The Na^+^, K^+^, and Ca^2+^ contents in the samples were determined by spectrophotometry with diagnostic kits as per the procedure mentioned by Labcare Diagnostics Pvt. Ltd., India.

### Estimates of TGFβ Levels in the Retinal Layers Using the ELISA

Commercially available and precoated ELISA kits (Elabscience, USA) were used for the *in vitro* quantitative determination of rat TGFβ concentrations in the retinal homogenates as per the manufacturer's instructions.

### Statistical Analysis

All statistical analyses were performed using GraphPad Instat® (version 5.0) software, and the results were expressed as means ± standard errors of the mean (SEMs). A one-way ANOVA, followed by the Newmann–Keuls test, was used for comparing the means of the different groups; similarly, a two-way ANOVA, followed by Bonferroni's test, was used for analyzing the data. The criterion for statistical significance was set at the ^*^p < 0.05, ^**^p < 0.01, and ^***^p < 0.001.

## Results

### Effects on IOP

**Figure 1:**
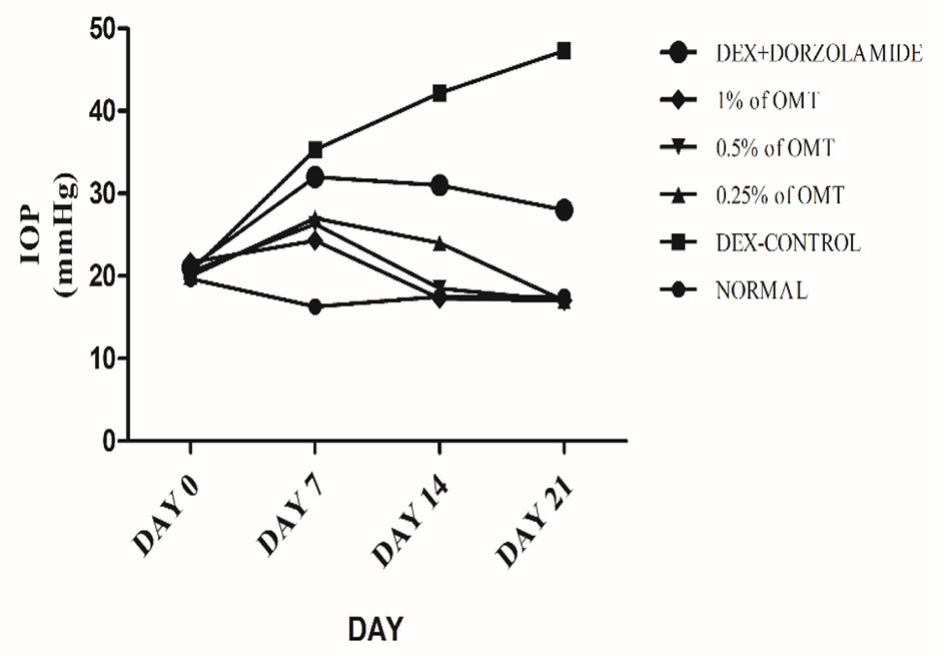
Effects over time on the IOP. Values are expressed as mean (*n* = 6). Data were analyzed by two-way ANOVA followed by Bonferroni's *post hoc* test. Normal: 100 μl of distilled water; control: 0.1 ml of 0.1% DEX solution; treatment 1: 50 μl of 0.25% OMT solution; treatment 2: 50 μl of 0.5% OMT solution; treatment 3: 50 μl of 1% OMT solution; standard: 100 μl of 1% of dorzolamide solution.

The experimental rats were regularly checked, with the help of the Tono-Pen tonometer (Reichert Technologies, Depew, USA), for the degree of IOP generated in the eyes. The IOP changes were recorded as the means of weekly changes and are represented in Table 1 and Figure 1.

**Table 1:** Effect of OMT on the IOP.

**Groups**	**Average IOP (mmHg)**			
	**Day zero**	**Day seven**	**Day fourteen**	**Day twenty-one**
100 μl of distilled water (Normal)	19.67 ± 1.35	16.33 ± 0.66	17.50 ± 1.33	17.50 ± 1.33
100 μl of 0.1% DEX solution (DEX-induced glaucoma control)	20.50 ± 0.76	35.33 ± 0.42^c^	42.17 ± 0.79^c^	47.33 ± 0.667^c^
50 μl of 0.25% OMT solution (treatment 1)	20.33 ± 1.05	27.17 ± 1.47^c^^e^	24.33 ± 1.47^b^^f^^i^	17.83 ± 0.70^f^^i^
50 μl of 0.5% OMT solution (treatment 2)	20.50 ± 1.11	26.33 ± 2.70^c^^e^	18.50 ± 0.76^f^^i^	17.00 ± 0.57^f^^i^
50 μl of 1% OMT solution (treatment 3)	21.67 ± 1.62	24.33 ± 1.76^b^^f^^g^	17.33 ± 0.66^f^^i^	17.00 ± 0.57^f^^i^
100 μl of the commercial dose of dorzolamide solution (standard)	21.00 ± 0.73	32.00 ± 1.67^c^	31.17 ± 1.81^c^^f^	28.83 ± 1.13^c^^f^

*Values are expressed as means ± SEMs (n = 6). Data were analyzed using the two-way ANOVA, followed by Bonferroni's post hoc test and are expressed as*

a *p < 0.05*,

b *p < 0.01, and*

c *p < 0.001 when compared to the normal group*,

d *p < 0.05*,

e *p < 0.01, and*

f *p < 0.001 when compared to glaucoma control group, and*

g *p < 0.05*,

h *p < 0.01, and*

i *p < 0.001 when compared to the standard group. The values are expressed as non-significant values*.

a, d, h *All the three represent comparisons made to the other groups with the normal group*.

Administration of DEX to the animals in the glaucoma control group, as compared to the normal group, produced a significant (*p*< *0.001*) elevation in the IOP approximately from the seventh day to the end of the experimental protocol. Topical administration of OMT at a 50-μl dose of 0.25, 0.5, or 1% notably inhibited the advance of IOP as compared to DEX-induced glaucoma control group and the standard group, (*p*< *0.001* and *p*< *0.001)*, respectively, in a time-dependent manner. After seven days of topical OMT administration at doses of 50 μl of 0.25% and 50 μl of 0.5%, a significant fall in the IOP was noted (*p* < 0.001) in comparison with the control group, whereas the group receiving treatment 3, that is, the group treated with 50 μl of 1% OMT, showed the best results in controlling the IOP in comparison with both the control and the standard groups, *p* < 0.001 and *p* < 0.01, respectively. From the 14 day to the 21st day of OMT administration, all three doses exhibited excellent control of the IOP, along with concurrent normalization of the IOP, when compared to the control and the standard groups, *p* < 0.001 and *p* < 0.001, respectively.

### Effects on Oxidative Stress Markers in Retinal Extracts

#### Effects on Antioxidants

**Figure 2:**
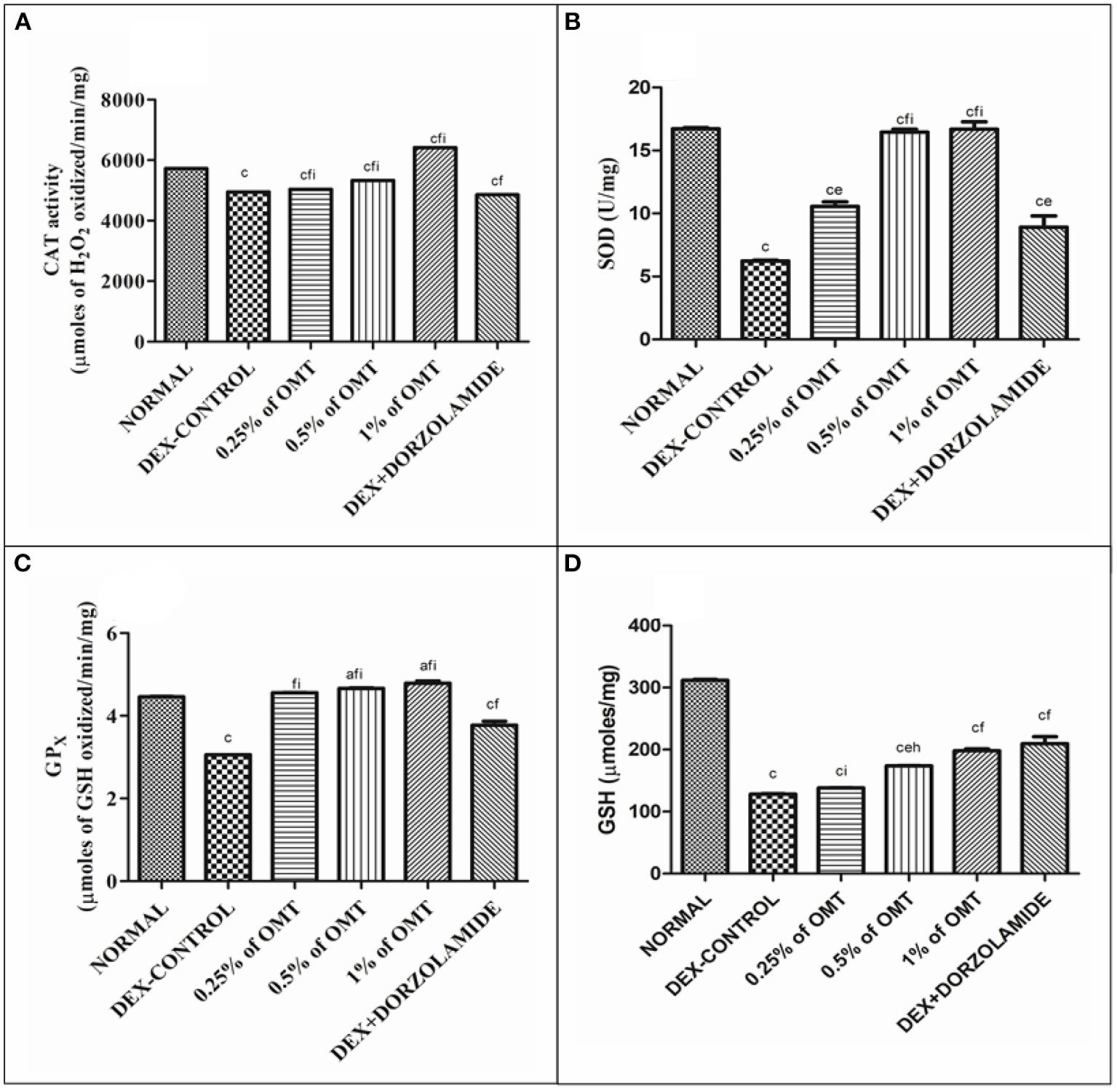
Effects of various treatments of OMT on the levels of antioxidants: **(A)** CAT, **(B)** SOD, **(C)** GPX, and **(D)** GSH. Values are expressed as mean ± SEM (n = 6). Data were analyzed by one-way ANOVA and are expressed as ap < 0.05, bp < 0.01, cp < 0.001 when compared to normal group, dp < 0.05, ep < 0.01, fp < 0.001 when compared to DEX-induced glaucoma control group, and gp < 0.05, hp < 0.01, and ip < 0.001 when compared to standard group, respectively. Normal: 100 μl of distilled water; control: 0.1 ml of 0.1% DEX solution; treatment 1: 50 μl of 0.25% OMT solution; treatment 2: 50 μl of 0.5% OMT solution; treatment 3: 50 μl of 1% OMT solution; standard: 100 μl of 1% of dorzolamide solution.

In the DEX-induced glaucoma control group, the levels of antioxidants, such as CAT, SOD, GPx, and GSH, were found to be decreased notably as compared to the normal group (*p* < 0.001) (Figures 2A–D). Subsequently, treatment with various doses of OMT resulted in a significant buildup in the levels of retinal antioxidants in contrast to the glaucoma control group. Two weeks of OMT administration at a dose of 50 μl of 0.25% OMT led to significant increases in some of the antioxidant levels, such as CAT (*p* < 0.001*, p* < 0.001*, p* <0.001) and SOD (*p* < 0.001, *p* < 0.01*, p* < 0.001) when compared to the normal, glaucoma control, and standard groups, respectively, GPx (*p* < 0.001*, p* < 0.001) when compared to the glaucoma control and the standard groups, respectively, and GSH (*p* < 0.001*, p* < 0.001) when compared to the normal and standard groups, respectively, but with no significant variations from the glaucoma control group. The administration of 50 μl of 0.5% OMT led to marked increase in the antioxidant levels of CAT (*p* < 0.001*, p* < 0.001*, p* < 0.001), SOD (*p* < 0.001*, p* < 0.001*, p* < 0.001), GPx (*p* < 0.05*, p* < 0.001), and GSH (*p* < 0.001*, p* < 0.01*, p* < 0.01) when compared to the normal, glaucoma control, and standard groups, respectively. The administration of the highest dose of treatment, that is, 50 μl of 1% OMT, produced the most notable rises in the levels of the antioxidants in the retinal layers: CAT (*p* < 0.001*, p* < 0.001*, p* < 0.001), SOD (*p* < 0.001*, p* < 0.001*, p* < 0.001), and GPx (*p* < 0.05*, p* < 0.001, *p* < 0.001) when compared to the normal, glaucoma control, and standard groups, respectively, and GSH (*p* < 0.001*, p* < 0.001) when compared to the normal and glaucoma control groups, respectively with no significant variations from the standard group.

#### Effects of Various Treatments on Lipid Peroxidation and Nitrite Content in the Retinal Layers

**Figure 3:**
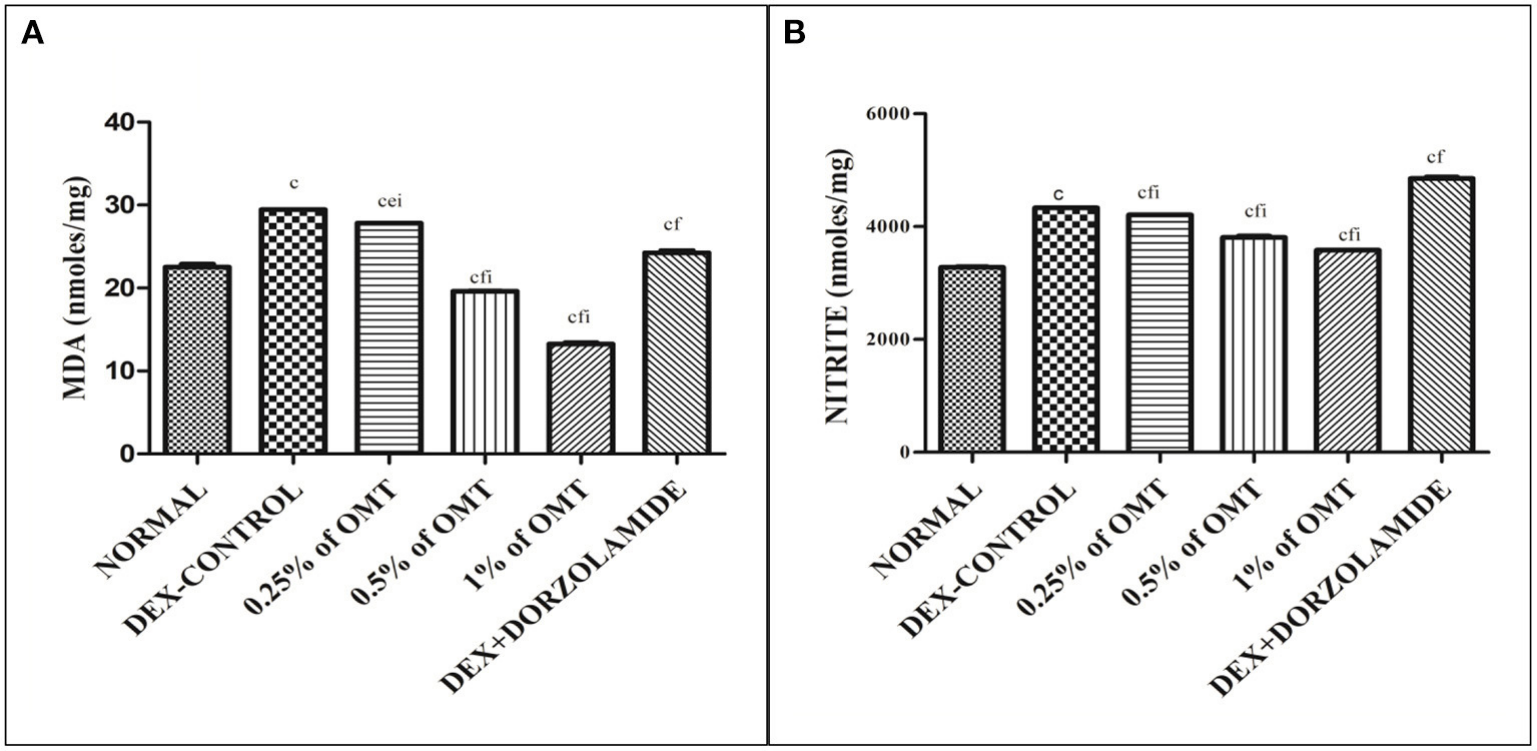
Effects of various treatments on lipid peroxidation and nitrite content in the retinal extracts: **(A)** MDA and **(B)** nitrite. Values are expressed as mean ± SEM (*n* = 6). Data were analyzed by one-way ANOVA and are expressed as ^a^*p* < 0.05, ^b^*p* < 0.01, ^c^*p* < 0.001 when compared to normal group, ^d^*p* < 0.05, ^e^*p* < 0.01, ^f^*p* < 0.001 when compared to DEX-induced glaucoma control group, and ^g^*p* < 0.05, ^h^*p* < 0.01, ^i^*p* < 0.001 when compared to standard group, respectively. Normal: 100 μl of distilled water; control: 0.1 ml of 0.1% DEX solution; treatment 1: 50 μl of 0.25% OMT solution; treatment 2: 50 μl of 0.5% OMT solution; treatment 3: 50 μl of 1% OMT solution; standard: 100 μl of 1% of dorzolamide solution.

The results (Figures 3A,B) demonstrate that the DEX control group significantly (*p* < 0.001) increased the MDA and nitrite content in the retina as compared to the normal group. The two weeks of OMT administration at the dose of 50 μl of 0.25% OMT led to a significant slash in the levels of lipid peroxidation quantified by the levels of MDA (*p* < 0.001*, p* < 0.01*, p* < 0.001) and nitrite (*p* < 0.001*, p* < 0.001*, p* < 0.001) as compared to the normal group, glaucoma control, and standard groups, respectively. 50μl of 0.5% OMT administration showed marked reduction in the levels of MDA (*p* < 0.001*, p* < 0.001*, p* < 0.001) and nitrite (*p* < 0.001*, p* < 0.001*, p* < 0.001) as compared to the normal group, glaucoma control, and standard groups, respectively. The highest dose of treatment, that is, 50μl of 1% OMT, exhibited the most notable decline in the levels of MDA (*p* < 0.001, *p* < 0.001*, p* < 0.001) and nitrite (*p* < 0.001*, p* < 0.001*, p* < 0.001) when compared to the normal group, glaucoma control, and standard groups, respectively. The standard group also displayed significant decline in the MDA levels (*p* < 0.001*, p* < 0.001) while a large increase exhibited in the nitrite concentrations (*p* < 0.001*, p* < 0.001) in the retinal layers when compared to the normal and glaucoma control groups, respectively.

### Effects of Various Treatments on Retinal Protein Contents

**Figure 4:**
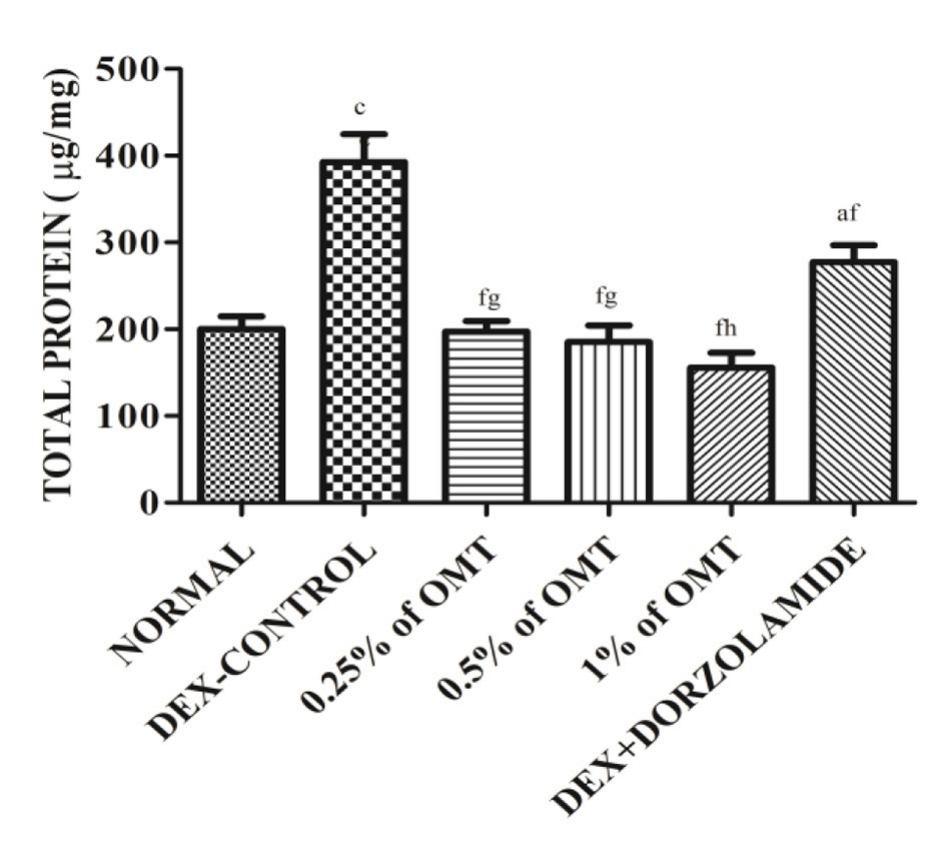
Effects of various treatments on the retinal protein extracts. Values are expressed as mean ± SEM (*n* = 6). Data were analyzed by one-way ANOVA and are expressed as ^a^*p* < 0.05, ^b^*p* < 0.01, ^c^*p* < 0.001 when compared to normal group, ^d^*p* < 0.05, ^e^*p* < 0.01, ^f^*p* < 0.001 when compared to DEX-induced glaucoma control group, and ^g^*p* < 0.05, ^h^*p* < 0.01, ^i^*p* < 0.001 when compared to standard group, respectively. Normal: 100 μl of distilled water; control: 0.1 ml of 0.1% DEX solution; treatment 1: 50 μl of 0.25% OMT solution; treatment 2: 50 μl of 0.5% OMT solution; treatment 3: 50 μl of 1% OMT solution; standard: 100 μl of 1% of dorzolamide solution.

The results (Figure 4) exhibit the remarkable remodeling of the total protein content in the retinal layers of the various experimental groups. The DEX control group significantly (*p* < 0.001) increased the total protein content in the lens as compared to the normal group. The two weeks of OMT administration at the dose of 50 μl of 0.25% OMT and 50 μl of 0.5% OMT in each of the respective experimental groups lead to a notable plunge in the levels of the total protein content (*p* < 0.001*, p* < 0.05) of the retinal layers, whereas treatment with 50 μl of 1% OMT led to an even more prominent decline in the protein content (*p* < 0.001, *p* < 0.01) as compared to the glaucoma control and standard groups respectively with no significant variation from the normal group. The standard group, however, raised the bar of the total protein concentration in the retinal layers (*p* < 0.05*)* when compared to the normal group but managed to reduce it significantly (*p* < 0.001*)* when compared to the glaucoma control group.

### Effects of Various Treatments on ATPase Activity in the Retinal Extracts

**Figure 5:**
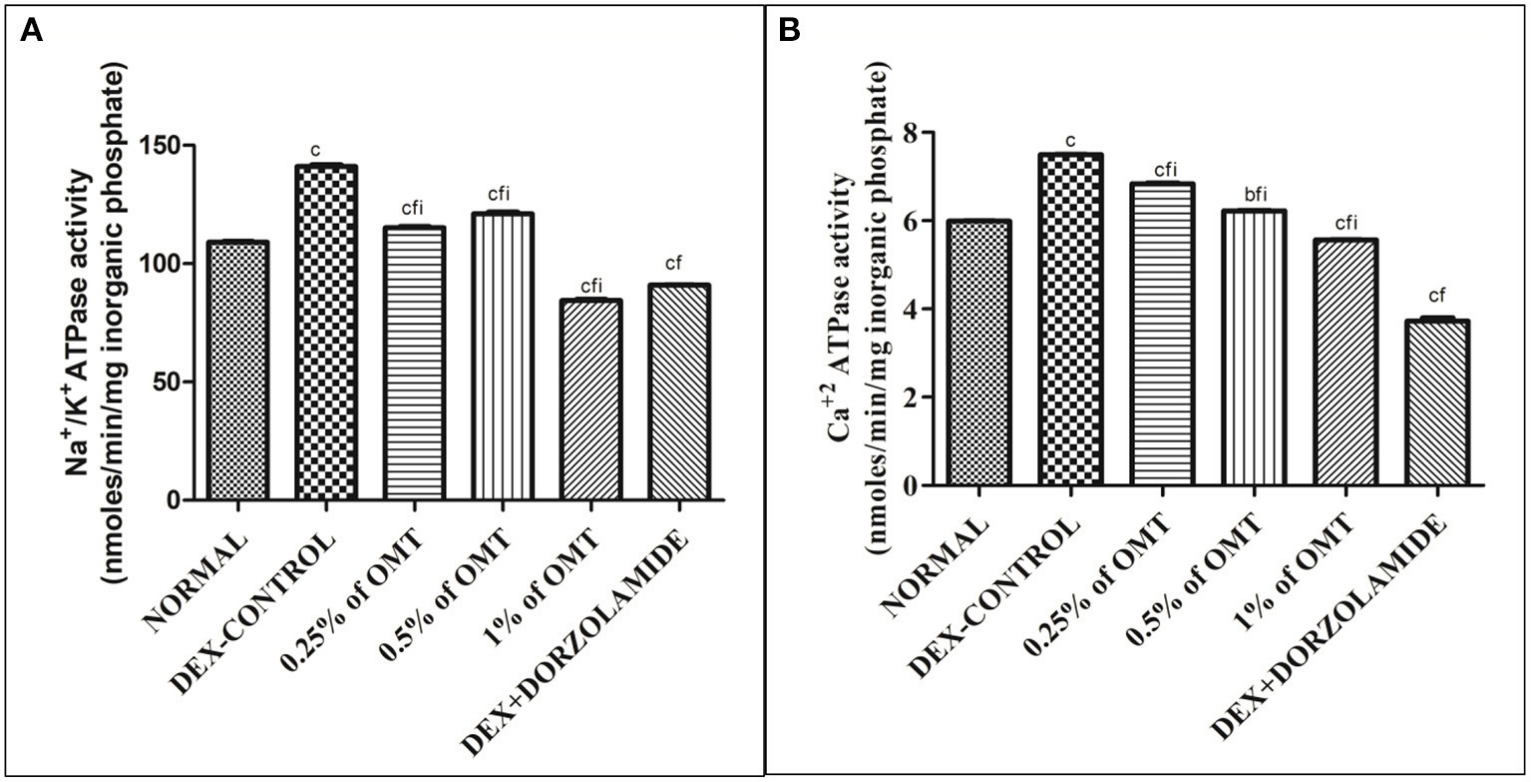
Effects of various treatments on the ATPase activity in the retinal layers: **(A)** Na^+^/K^+^ ATPase and **(B)** Ca^+2^ ATPase. Values are expressed as mean ± SEM (*n* = 6). Data were analyzed by one-way ANOVA and are expressed as ^a^*p* < 0.05, ^b^*p* < 0.01, and ^c^*p* < 0.001 when compared to normal group, ^d^*p* < 0.05, ^e^*p* < 0.01, and ^f^*p* < 0.001 when compared to DEX-induced glaucoma control group, and ^g^*p* < 0.05, ^h^*p* < 0.01, and ^i^*p* < 0.001 when compared to standard group, respectively. Normal: 100 μl of distilled water; control: 0.1 ml of 0.1% DEX solution; treatment 1: 50 μl of 0.25% OMT solution; treatment 2: 50 μl of 0.5% OMT solution; treatment 3: 50 μl of 1% OMT solution; standard: 100 μl of 1% of dorzolamide solution.

Figures 5A,B reveal that the animals in the DEX control group had significantly (*p* < 0.001) increased Na^+^/K^+^ ATPase in their retinal layers, as compared to the normal group. Two weeks of OMT administration at a dose of 50 μl of 0.25% OMT led to a significant reduction in the levels of Na^+^/K^+^ ATPase (*p* < 0.001, *p* < 0.001, *p* < 0.001) and Ca^2+^ATPase (*p* < 0.001, *p* < 0.001, *p* < 0.001) as compared to the normal, glaucoma control, and standard groups, respectively. Administration of 50 μl of 0.5% OMT led to an even more notable decline in the levels of Na^+^/K^+^ ATPase (*p* < 0.001, *p* < 0.001, *p* < 0.001) and Ca^2+^ATPase (*p* < 0.01, *p* < 0.001, *p* < 0.001), as compared to the normal, glaucoma control, and standard groups, respectively. The treatment with the highest dose, that is, 50 μl of 1% OMT, led to the largest declines in the levels of Na^+^/K^+^ ATPase (*p* < 0.001, *p* < 0.001, *p* < 0.001) and Ca^2+^ATPase (*p* < 0.001, *p* < 0.001, *p* < 0.001), as compared to the glaucoma control and standard groups, respectively. In the standard group, the levels of Na^+^/K^+^ ATPase and Ca^2+^ATPase (*p* < 0.001, *p* < 0.001*)* were evidently reduced, as compared to the normal and glaucoma control groups, respectively.

### Effects of Various Treatments on Na^+^, K^+^, Ca^+2^ ion Activities in the Retinal Extracts

**Figure 6:**
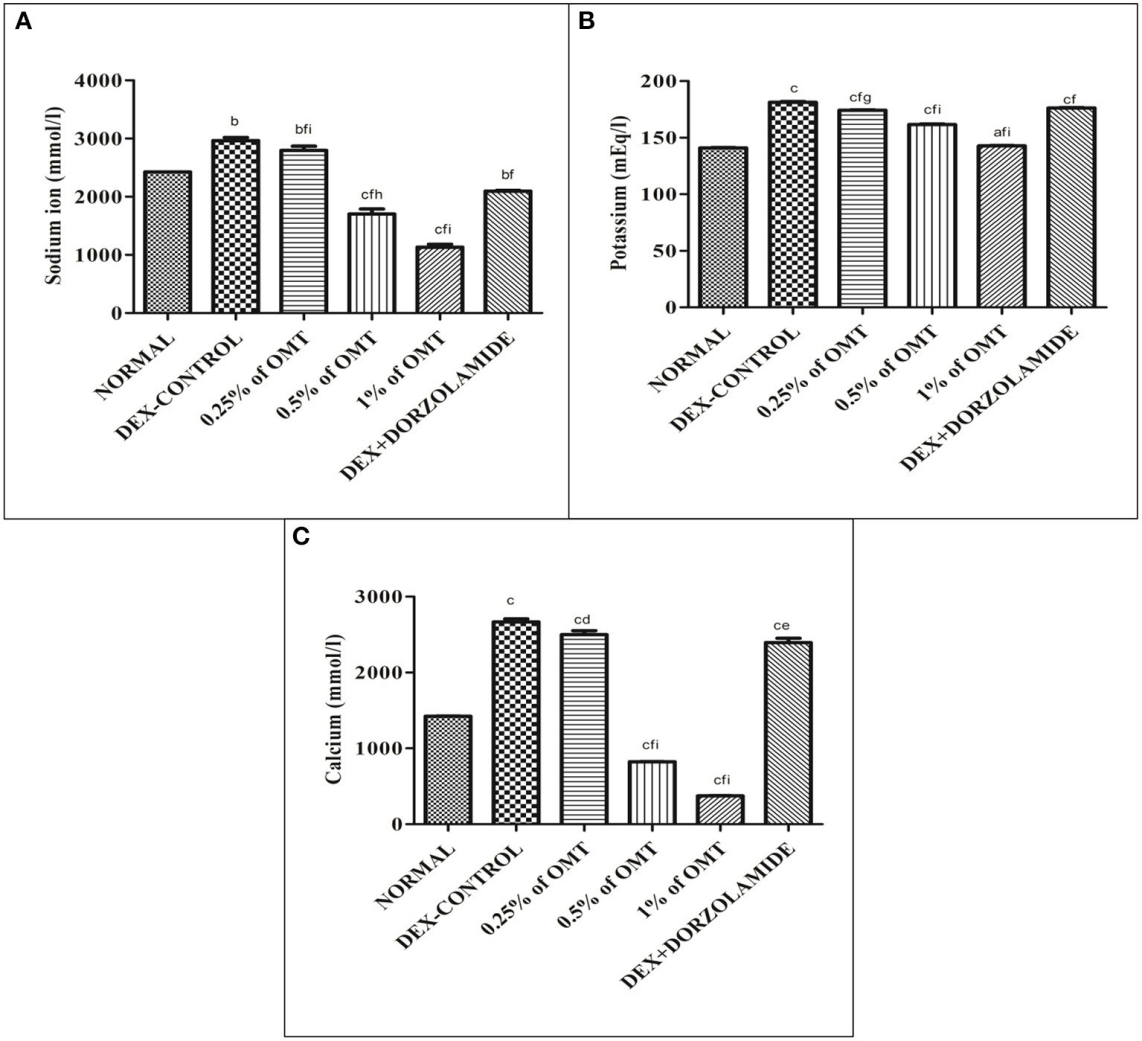
Effects of various treatments on **(A)** Na^+^, **(B)** K^+^, **(C)** Ca^+2^ ion activities in the retinal extracts. Values are expressed as mean ± SEM (*n* = 6). Data were analyzed by one-way ANOVA and are expressed as ^a^*p* < 0.05, ^b^*p* < 0.01, ^c^*p* < 0.001 when compared to normal group, ^d^*p* < 0.05, ^e^*p* < 0.01, ^f^*p* < 0.001 when compared to DEX-induced glaucoma control group, and ^g^*p* < 0.05, ^h^*p* < 0.01, ^i^*p* < 0.001 when compared to standard group, respectively. Normal: 100 μl of distilled water; control: 0.1 ml of 0.1% DEX solution; treatment 1: 50 μl of 0.25% OMT solution; treatment 2: 50 μl of 0.5% OMT solution; treatment 3: 50 μl of 1% OMT solution; standard: 100 μl of 1% of dorzolamide solution.

Figures 6A–C reveal that the animals in the DEX control group had significantly increased Na^+^ (*p* < 0.01), K^+^ (*p* < 0.001), and Ca^+2^ (*p* < 0.001) in their retinal layers, as compared to the normal group, respectively. Two weeks of OMT administration at a dose of 50 μl of 0.25% OMT led to a significant reduction in the levels of Na^+^ (*p* < 0.01*, p* < 0.001*, p* < 0.001), K^+^ (*p* < 0.001, *p* < 0.05, *p* < 0.001), and Ca^2+^ (*p* < 0.001, *p* < 0.05), as compared to the normal, glaucoma control, and standard groups, respectively. The group receiving 50 μl of 0.5% OMT recorded remarkable drops in the levels of Na^+^ (*p* < 0.001, *p* < 0.001, *p* < 0.01), K^+^ (*p* < 0.001, *p* < 0.001, *p* < 0.001), and Ca^2+^ (*p* < 0.001, *p* < 0.001, *p* < 0.001), as compared to the normal, glaucoma control, and standard groups, respectively. The highest dose of treatment, that is, 50 μl of 1% OMT, led to the most notable decline in the levels of Na^+^ (*p* < 0.001, *p* < 0.001, *p* < 0.001), K^+^ (*p* < 0.05, *p* < 0.001, *p* < 0.001), and Ca^2+^ (*p* < 0.001, *p* < 0.001, *p* < 0.001), as compared to the glaucoma control and standard groups, respectively.

### Effects of OMT on the Expression of TGFβ1

**Figure 7:**
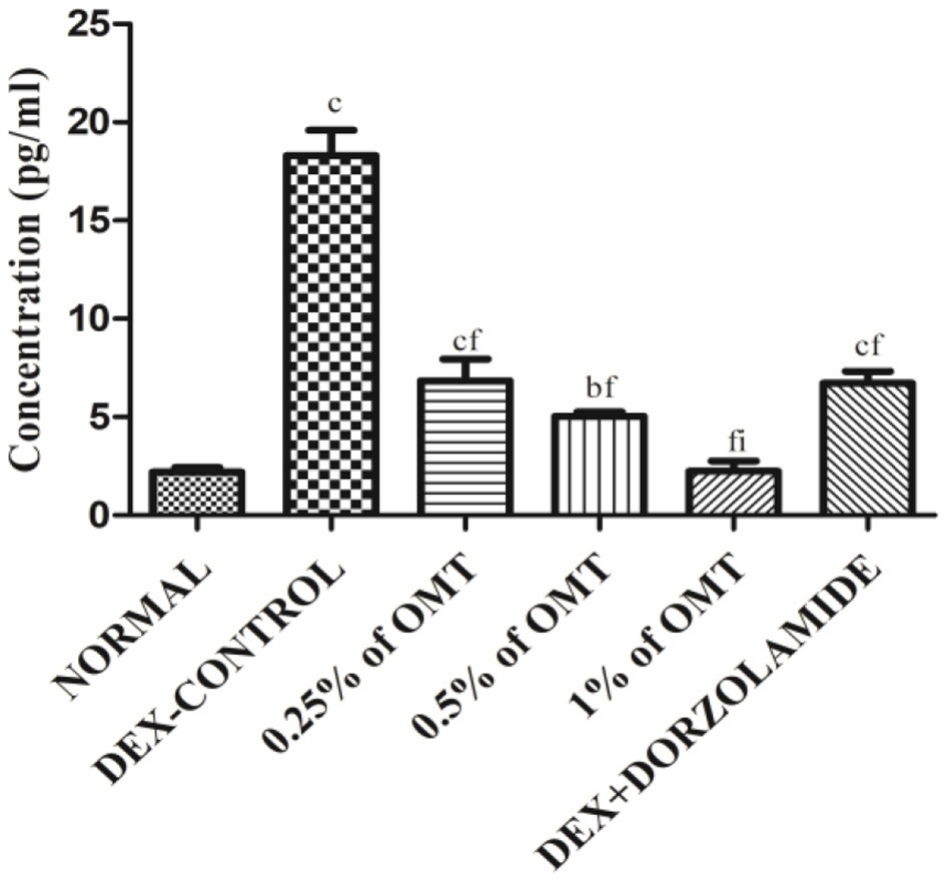
Effects of OMT on the expression of TGFβ1. Values are expressed as mean ± SEM (*n* = 6). Data were analyzed by one-way ANOVA and are expressed as ^a^*p* < 0.05, ^b^*p* < 0.01, ^c^*p* < 0.001 when compared to normal group, ^d^*p* < 0.05, ^e^*p* < 0.01, ^f^*p* < 0.001 when compared to DEX-induced glaucoma control group, and ^g^*p* < 0.05, ^h^*p* < 0.01, ^i^*p* < 0.001 when compared to standard group, respectively. Normal: 100 μl of distilled water; control: 0.1 ml of 0.1% DEX solution; treatment 1: 50 μl of 0.25% OMT solution; treatment 2: 50 μl of 0.5% OMT solution; treatment 3: 50 μl of 1% OMT solution; standard: 100 μl of 1% of dorzolamide solution.

The animals in the DEX control group exhibited gigantic rises (*p* < 0.001) in the expression of TGFβ1 in their retinal layers, as compared to the normal group while dose-dependent decline in the expressions of TGFβ1 was observed in the various groups of animals treated with OMT (Figure 7). The animals that received a dose of 50 μl of 0.25% OMT demonstrated a notable inhibition (*p* < 0.001) when compared to the glaucoma control group, but had significantly higher expression (*p* < 0.001) when compared to the normal group; the animals that received a dose of 50 μl of 0.5% OMT exhibited sizable declines (*p* < 0.001) when compared to the control group, but failed to achieve normal levels (*p* < 0.01). The experimental animals that were treated with a dose of 50 μl of 1% OMT demonstrated the most notable fall in the TGFβ1 levels (*p* < 0.001*, p* < 0.001) when compared to the glaucoma control and standard groups, respectively. The most astonishing feature in the group of animals receiving 50 μl of 1% OMT was the restoration of normal levels of TGFβ1 in the retinal layers, which points to rehabilitation of homeostasis using OMT against dorzolamide, which was unable to restore normal levels of TGFβ1 in the retinal layers.

### Effects of OMT on the Retinal Structure and Visual Field

**Figure 8:**
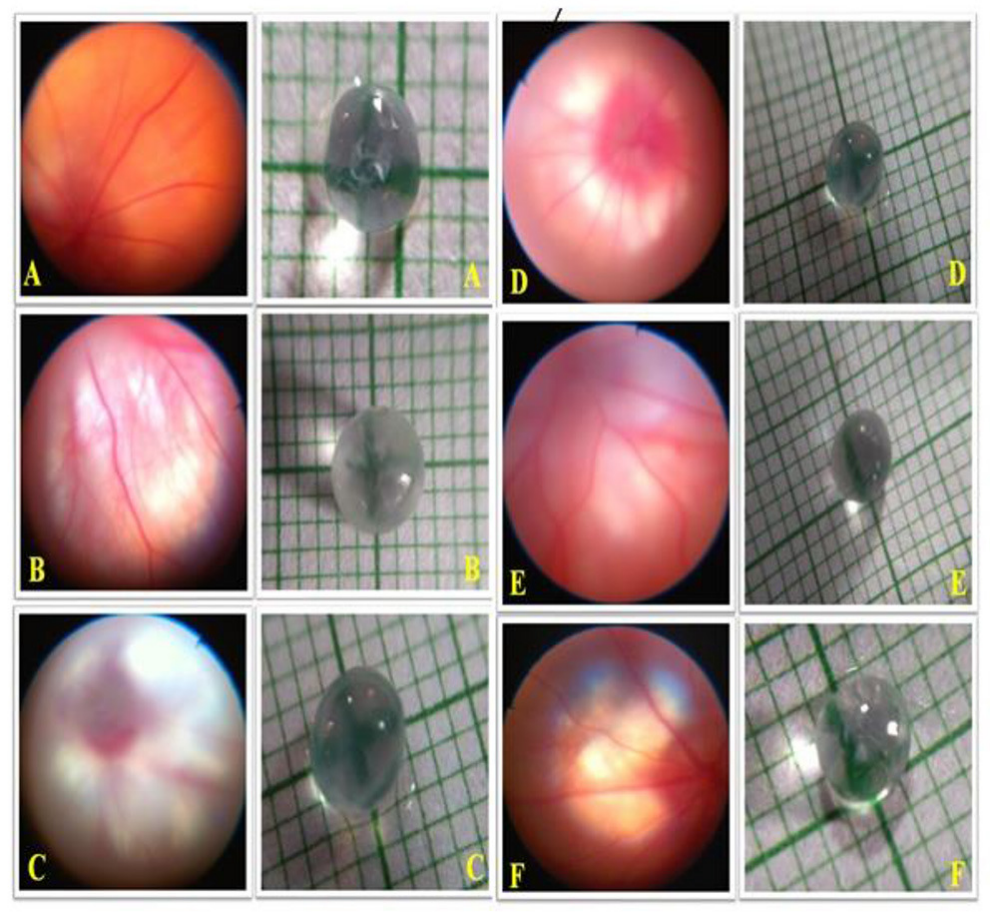
Effects of OMT on the retinal structure and visual field. Normal group: **(A)** normal retinal vasculature along with normal visual field as indicated by lens opacity; **(B)** glaucoma control group: evident retinal fibrosis along with bilateral opacity in the lens upon DEX exposure; **(C)** standard group: restoration of retinal vasculature, however, without efficient detangling of the blood vessels and reduction in opacity as compared to the control group; **(D)** treatment 1 group: retinal fibrosis still evident with opacity in the lens; **(E)** treatment 2 group: significant restoration of retinal vasculature along with betterment in the visual field as indicated by the decline in lens opacity; **(F)** treatment 3 group: proper restoration of the retinal vasculature along with optimum lens clarity. Normal: 100 μl of distilled water; control: 0.1 ml of 0.1% DEX solution; treatment 1: 50 μl of 0.25% OMT solution; treatment 2: 50 μl of 0.5% OMT solution; treatment 3: 50 μl of 1% OMT solution; standard: 100 μl of 1% of dorzolamide solution.

The animals in the DEX-induced group exhibited a deposited fibrous network in the retinal vasculature, along with an increase in the lenticular opacity. The animals receiving 0.25% of OMT showed slight declines in amounts of the deposited fibrous network and in the lenticular opacity, as compared to the glaucoma control group whereas the group receiving 0.5% of OMT showed significant falls in amounts of deposited fibrous network deposition and subsequent increased clarity of the lens, as compared to the glaucoma control and standard group. The group receiving 1% of OMT exhibited complete restoration of the retinal vasculature, along with significant clarity of the lens, as compared to the glaucoma control and standard group (Figure 8).

## Discussion

Steroids are a class of antiinflammatory agents that find use in several systemic manifestations. Overindulgence in the application of topical or systemic steroids, however, is known to contribute to glaucomatous conditions, which can be detected by an increase in the IOP. Steroids, such as DEX, prednisolone, and others, are known to alter the ECM in ocular tissues *via* various pathways (7, 17, 27, 33–36). Inhibition of lysosomal hyaluronidase by steroids and the resultant activation of TGFβ is the key to retarding ECM degradation and enhancing fibrillar materials in the ocular vasculature with further benefaction into biological edema following a decline in the radius of the TM, thus causing an increase in the IOP (7, 17, 33, 36).

In this study, DEX (0.1%) was topically administered to the animals in the glaucoma control group four times daily at a dose of 0.1 ml for 21 days, and a significant hike in the IOP was noted, which is consistent with the results of studies previously conducted on humans and animals (5, 17, 34, 35). Diverse mechanisms have been used to explain the amplification of IOP by DEX. DEX increases the concentrations of thrombospondin I, integrin, laminin, and fibronectin, all of which happen to activate TGFβ signaling in ocular tissues, resulting in persistent fibrosis *via* cell–matrix interactions (7, 17, 27). In addition to its effects on the ECM, DEX can induce the activation of the endoplasmic reticulum and Golgi apparatus, finally boosting the DNA content in the TM. Modeling new channel debris due to reduced phagocytic activities at the TM further results in delayed clearance of the ocular vasculature, thus finally giving a clear picture of IOP enhancement (33, 36). The DNA damage caused by DEX administration enhances the release of reactive oxygen species (ROS) that further function in the activation and signaling of TGFβ (17, 37).

Dexamethasone administration in glaucomatous rats resulted in a steady amplification of the IOP whereas OMT administration resulted in a significant decline in and consequent normalization of the same. The treatment groups achieved IOP values similar to that found in the normal group, almost diminishing the effects of DEX; although the standard treatment did help reduce the IOP compared to the control group, it could not normalize the IOP value, thus clearly indicating the increased efficacy of OMT treatment when compared to dorzolamide treatment, even though the dorzolamide administered was a commercial preparation formulated with penetration enhancers. IOP regulation, however, is not the only requisite for inhibiting the progression of glaucoma; hence, biochemical parameters and histopathology were investigated to determine the safest and most effective dose of OMT to inhibit the progression of glaucoma.

The activation of TGFβ, especially TGFβ1, leads to the concurrent downregulation of endogenous antioxidants such as GSH, SOD, GP_X_, CAT, and ascorbic acid *via* a significant inhibitory impact on the mitochondrial membrane potential while simultaneously enhancing the ROS production that fosters an immediate reduction in the protective mechanisms of the TM cells and facilitates damage to the optic nerve disc and astrocytes (15, 19, 38, 39). All these effects that induced during the progression of glaucoma by DEX denote the withdrawal of the salubrious impacts of the antioxidant system and, thus, clearly define the role of DEX in TGFβ activation and the emergence of glaucoma in the glaucoma control group (40–45).

These studies denote the beneficial impacts of OMT administration on the glaucomatous experimental rats. OMT administration enhances the effects of various antioxidant systems, such as CAT, SOD, GP_X_, and reduced GSH, present in the retinal layers while significantly delaying and reducing the rates of lipid peroxidation, as denoted by the decline in the MDA and the nitrite levels. Dorzolamide, being the standard drug candidate in this study, has been observed to heighten the nitrite levels, which is due to its effects on vasodilation mediated *via* the evolution of nitric oxide from nitrite. Dexamethasone, primarily being an antiinflammatory agent, does not crucially affect the nitrite levels when compared to its effects on other pathological enhancers; however, OMT has still been noticed to lower the nitrite levels to retard the generation of nitric oxide, thus clarifying its effects as an antiinflammatory agent. Oxymatrine has been studied to include various toxic molecules generated by TGFβ activation, thus reducing the consumption of antioxidant systems and their consequent elevations in diseased tissues (46). The profound antioxidant effects exerted by OMT further help to delay the rates of lipid peroxidation *via* inhibition of the MDA and the nitrite contents (12, 47). The antioxidant ability of OMT can be attributed to its chemical structure in which its oxygen atoms readily combine with the hydroxyl radicals in the diseased cells and function as a free radical scavenger, thus increasing the concentration of antioxidant systems and consequently decreasing the number of the ROS-activating agents, further inhibiting the generation of an immune response (12).

The total protein concentration is a crucial part of the AH drainage facility and has been shown to be increased in concentration in the glaucoma control group, suggesting a disruption in the optimum flow of AH. Though none of the studies previously conducted have reported any relationship between OMT administration and the total protein content, studies have reported its efficacy in lowering the levels of matrix metalloproteinases (MMPs) and albumin, both of which are found to be upregulated in the retinal layers during glaucoma and are important regulators of the AH outflow facility (48, 49). Furthermore, TGFβ1 is known to elevate the levels of MMPs in the retinal layers and to provide systematically a distinct view of the relationship between TGFβ1 activation and the occurrence of fibrotic tangles (50). In this study, topical administration of OMT resulted in a profound reduction in the total protein concentration, thus emphasizing the antifibrotic effects of OMT, which has found a perfect role in decreasing the fibrogenic material deposited in the ECM (12).

Previously conducted studies have reported the effects of DEX in enhancing the Na^+^/K^+^ ATPase *via* an increase in the phosphorylation and subsequent endocytosis, thereby enhancing the pump function and increasing the AH production with an already depressed outflow facility, finally amplifying the IOP and the consequent increase in the intracellular concentrations of Na^+^ and K^+^ ions, resulting in an alteration of homeostasis (51). In this study, OMT administration was shown to depress the Na^+^/K^+^ ATPase activity, with a resultant decline in the related ionic concentrations in the retinal layers and a consequent normalization of activity, thereby shifting the focus to its underestimated roles in the regulation of pump functions and ionic balances. The extensive activities of Ca^+2^ ATPase in the progression of glaucoma have not yet been experimentally elucidated; however, significant rises in intracellular calcium levels and related neurodegeneration and oxidative stress have been previously demonstrated (18, 52). This study has set a milestone in establishing a factual basis for the Ca^+2^ ATPase activity, which is amplified upon glaucoma induction in the control group animal, thereby forcing an enhanced entry of calcium ions, which is responsible the increased levels of ROS and the resultant excitotoxicity of the RGCs (18, 52). OMT administration has been found to lower the Ca^+2^ ATPase activity significantly, thus pointing toward its actions in reducing the intracellular calcium content. The resultant calcium overload causes excessive contractility due to an amplified actin–myosin interaction, leading to the deposition of fibrogenic materials and structural alterations (53). OMT is responsible for the decline in the levels of the inward L-type calcium current, especially during conditions of calcium overload, and this study also remains in sync with previous considerations as OMT caused prominent reductions in the rates of calcium overload and enhanced the restoration of homeostasis (41).

The results obtained from the ELISA analyses of TGFβ1 clearly defined an enhancement of the TGFβ1 levels in the retinal samples of the DEX-induced glaucoma control group whereas consistent treatment with OMT led to a dose-dependent decline, which is known to inhibit TGFβ1 selectively, thus putting enough emphasis on the impact of TGFβ1 hyperactivation in glaucoma and its resultant reduction. The relationships between the various biochemical parameters and TGFβ1 have been formerly elucidated, and a distinct correlation can thus be portrayed.

Pictures of the retinal vasculature and lens obtained from the DEX-induced glaucoma control animals showed evident retinal fibrosis and bilateral opacity in the lens upon DEX exposure whereas the standard group exhibited restoration of retinal vasculature, but without an efficient disentanglement of the blood vessels and reduction in opacity as compared to the control group. The groups receiving OMT showed dose-dependent effects on the retinal vasculature and lenticular opacity. The animal treated with OMT manifested proper restoration of the retinal vasculature along with optimum lens clarity; this is consistent with the biochemical estimates showing that the group receiving the highest dose of treatment experienced maximum beneficial effects.

## Conclusion

**Figure 9:**
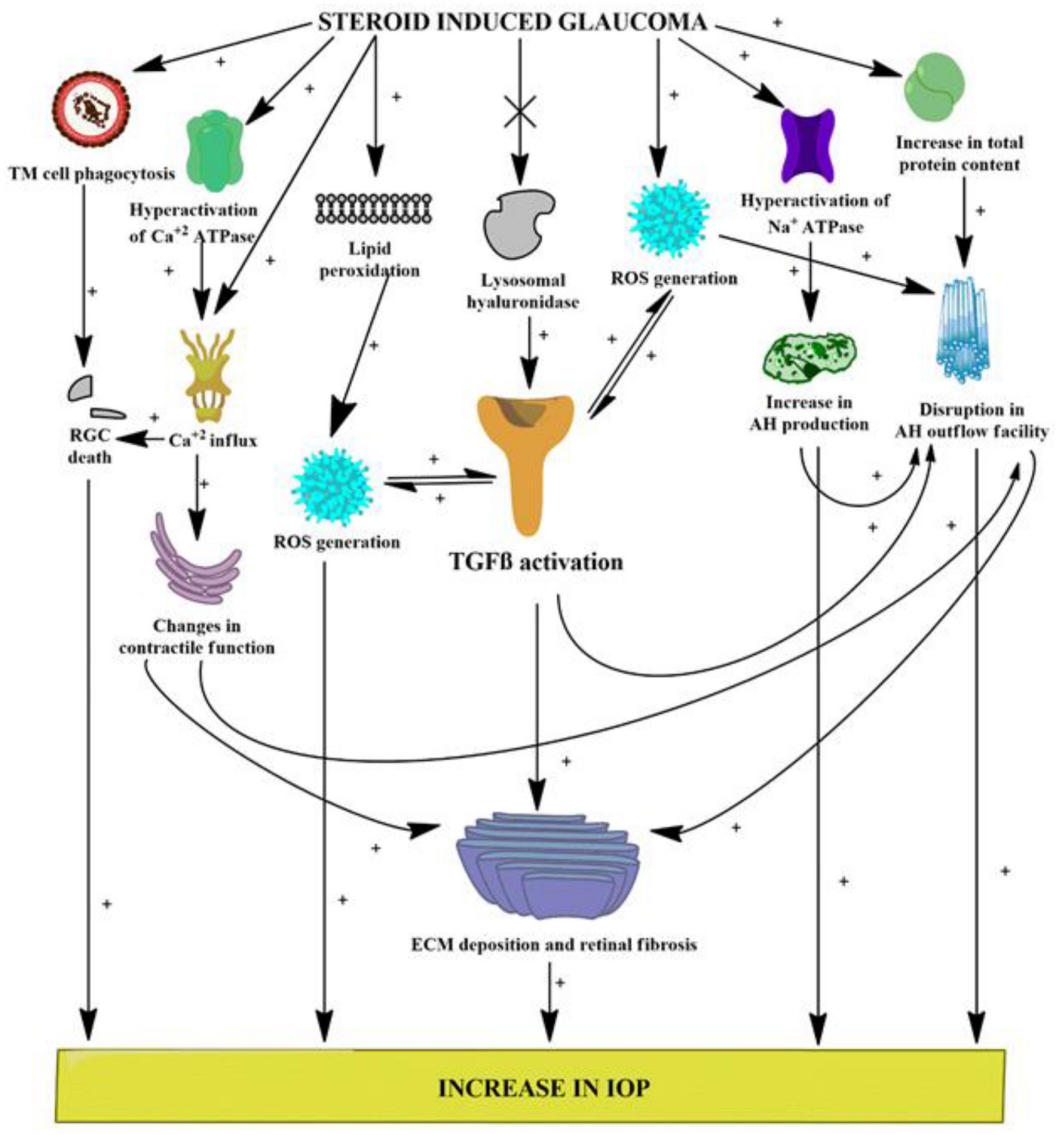
Various possible targets served by DEX administration leading to glaucomatous conditions. [(+) indicates activation or increase; (x) indicates inhibition or decrease]. ROS, reactive oxygen species; RGCs, retinal ganglion cells; AH, aqueous humor; IOP, intraocular pressure; TGFβ, transforming growth factor β; iNOS, inducible nitric oxide synthase.

**Figure 10:**
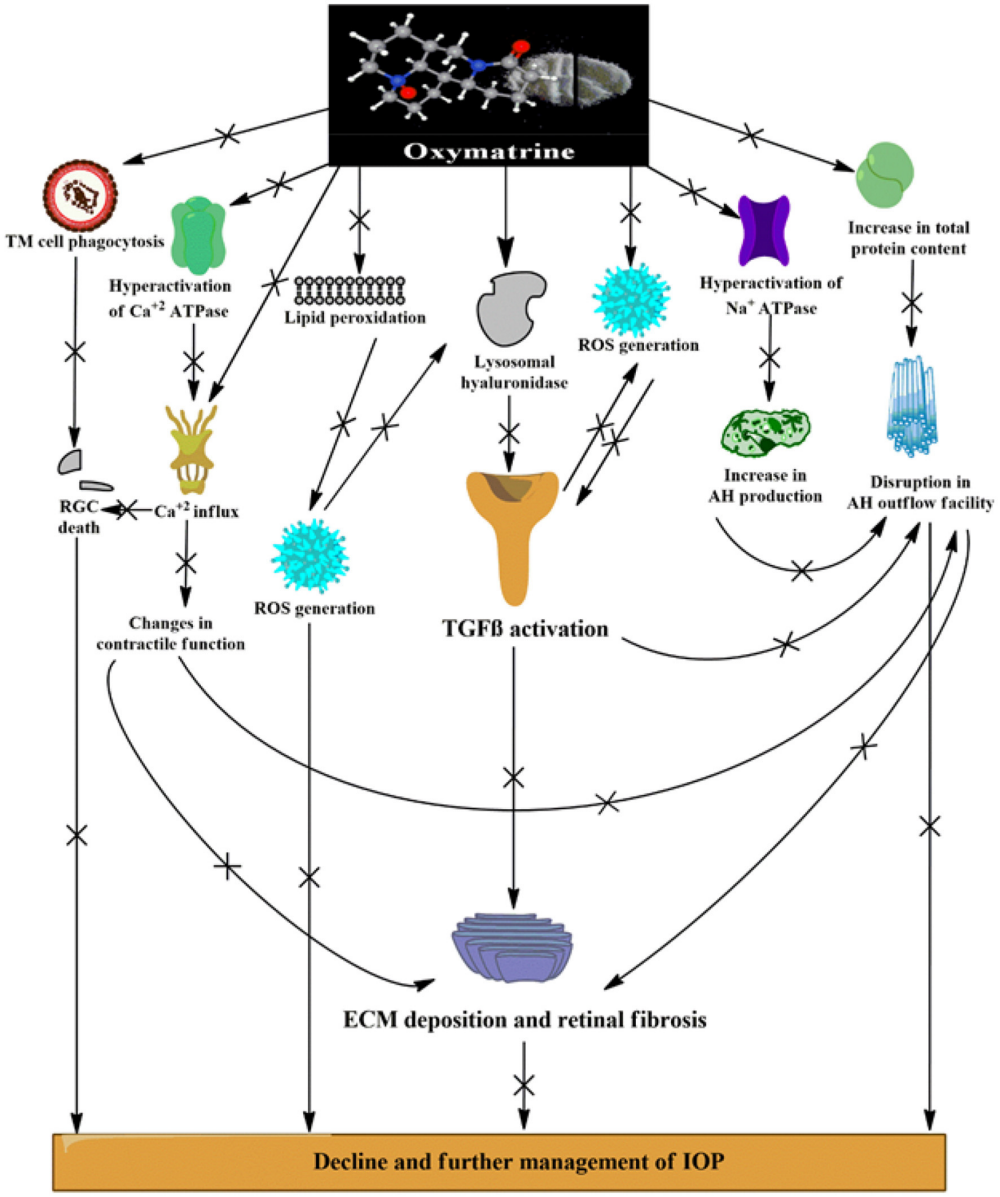
Various possible targets served by OMT administration leading to effective management of IOP and mechanobiological alterations induced by DEX. [(+) indicates activation or increase; (x) indicates inhibition or decrease]. ROS, reactive oxygen species; RGCs, retinal ganglion cells; AH, aqueous humor; IOP, intraocular pressure; TGFβ-transforming growth factor β; iNOS, inducible nitric oxide synthase.

The preclinical findings obtained from this study implicate an evident role of the TGFβ1 isoform in the progression of glaucoma, which might contribute to the various pathological alterations seen in patients with glaucoma (Figure 9). Elevations in oxidative stress, lipid peroxidation, nitrite level, and total protein content, inhibition of ECM-degrading enzymes, dysregulation in the pump activities and ionic levels, and alteration of the retinal vasculature *via* activation of TGFβ1 all work to fan the flames in the pathophysiology of glaucoma. The stipulated drug candidate used in this research, that is, OMT, was able to slow down the pathogenesis of glaucoma by not only lowering and further managing IOP but also substantially restoring the homeostasis of the retinal vasculature, which clearly identified the TGFβ1 isoform as a future target for therapies related to glaucoma inhibition (Figure 10).

## Data Availability Statement

The original contributions presented in the study are included in the article/supplementary material, further inquiries can be directed to the corresponding author/s.

## Ethics Statement

The animal study was reviewed and approved by Institutional Animal Ethics Committee (IAEC), Committee for the Purpose of Control and Supervision for Experiments on Animals (CPCSEA), Government of India.

## Author Contributions

AD, KPN, and SB framed the study. AD, OK, and AS conducted the *in vivo* experiments and experimental analysis. AD and SB wrote the manuscript. SB, KPN, and JS analyzed all the experimental data and proofread the manuscript. All authors contributed to the article and approved the submitted version.

## Conflict of Interest

The authors declare that the research was conducted in the absence of any commercial or financial relationships that could be construed as a potential conflict of interest.

## Publisher's Note

All claims expressed in this article are solely those of the authors and do not necessarily represent those of their affiliated organizations, or those of the publisher, the editors and the reviewers. Any product that may be evaluated in this article, or claim that may be made by its manufacturer, is not guaranteed or endorsed by the publisher.
